# Bridging Health Inequalities in the Learning Disability Population: A Quality Improvement Project on Weight Management

**DOI:** 10.1192/bjo.2025.10326

**Published:** 2025-06-20

**Authors:** Esraa Abdelrahman, Kainat Khan, Nicole Eady

**Affiliations:** East London NHS Foundation Trust, London, United Kingdom

## Abstract

**Aims:** People with learning disabilities (LD) experience significant health inequalities, including higher rates of obesity and associated comorbidities. This quality improvement (QI) project within East London NHS Foundation Trust (ELFT) aimed to enhance weight management interventions for individuals with LD by improving engagement with weight monitoring and lifestyle interventions.

**Methods:** A multidisciplinary team (MDT) implemented the Model for Improvement framework to address low rates of BMI (Body Mass Index) and weight recording among service users. Baseline data showed that less than 30% of service users had BMI recorded, and fewer than 3% had their weight documented at appointments. Interventions included training staff in routine weight monitoring, introducing accessible health education materials, and implementing structured weight management pathways. Plan-Do-Study-Act (PDSA) cycles were used to iteratively test and refine these changes. A total of 30 service users participated in the project over 12 months.

**Results:** The interventions led to significant improvements in weight monitoring and engagement with weight management strategies. By the end of the project, BMI recording increased to over 80%, and weight documentation rose to over 60%, demonstrating improved adherence to monitoring practices. In terms of clinical outcomes, 40% of service users achieved a ≥5% reduction in body weight, highlighting the effectiveness of tailored interventions. Furthermore, there is increased engagement with structured dietary and physical activity programmes, with over 75% of service users consistently participating. Additionally, 85% of carers reported increased confidence in supporting service users with weight management, further enhancing sustainability.

**Conclusion:** This QI project successfully demonstrated that structured, MDT-led interventions can improve weight management and health monitoring in individuals with LD. Increased documentation rates and service user engagement suggest that targeted, person-centred approaches can address health inequalities effectively. Future efforts will focus on scaling up these interventions, addressing remaining barriers to participation, and evaluating long-term sustainability.

